# The Great War: IX. Flies and Disease

**Published:** 1918-06-08

**Authors:** 


### THE GREAT WAR.

### IX.—Flies and Disease.

Every village in France suffers from a plague of flies during the summer months. The battle area swarmed with them in 1914 and 1915, in 1916 there were fewer, and in 1917 very few, the British crusade against them was beginning to show results. The commonest species are the *Musca domestica*, *Stomoxys*, blow-fly, and *Pycnosoma*, of these the *Musca*, or domestic fly, is the most numerous and the most important from a sanitary point of view.

### Musca Domestica.

The modern history of the house-fly as a disease carrier dates from the Spanish-American War of 1898, during which one-fifth of the American Army in the Philippines contracted enteric fever. The Board appointed by the United States Government to investigate the causes condemned the fly, which thus became an object of interest to the sanitarian, and eventually scientists on the Continent and at Home began to study the biology and habits of the newly recognised pest.

In 1908 the Local Government Board took the matter up, and three reports by the appointed research officers, Jepson, Graham Smith, Bernstein, Monckton-Copeman, and others, had already been published up to the outbreak of war. The fly pest at the front gave a great impetus to the subject, and in 1915-16 Professor Newstead of Liverpool University, Professor Austin of the British Museum, and Professor Jacks, entomologist to the South African Government, spent the fly seasons with the Army at the Front in order to study the conditions and watch the effects of remedies.

### Life and Development.

The house-fly lays its eggs, 100 to 150 in number, mainly in the excreta of animals. The eggs hatch out in about twenty-four hours, and the larvæ burrow. In from five to eight days the larvæ pupate sufficiently near the surface to allow easy escape of the imago, which, under favourable circumstances, takes place in three or four days. Flies become sexually mature in ten to fourteen days, and four days after mating they are potential egg-layers. The life of the female is approximately four layings at intervals of twelve days, or about two months. According to Hewitt the race is continued by the survival of a few hardy individuals which hibernate: there is no doubt, also, that the pupæ provide for the succession.

### Digestive System.

The most important part of the fly from a disease-spreading point of view is the alimentary canal. Flies feed on filth of all kinds, including excreta, and on the food of man, especially sugar, milk, cream, and cheese. The proboscis of the fly is adapted to a sucking function, and it cannot take in solid food; solids are first dissolved by the regurgitated contents of the crop, or if the crop is empty, by the saliva, of which there is an abundant supply from the long glands, which open by a single duct into the mouth. The mouth is connected with a powerful muscular pump—the pharyngeal pump—which pumps the food through the œsophagus into the crop and into the ventriculus. The crop serves as a reservoir for food; it enables the fly to obtain in a short time sufficient food to last for several days. The ventriculus is the chyle stomach in which digestion takes place. During feeding, the crop is first filled, within a minute, and afterwards the food passes directly into the ventriculus. After gorging themselves, flies move about, and at intervals sit still and regurgitate and reabsorb large drops of fluid from their crops several times, the purpose being either the mixing of food with saliva to assist digestion, or the solution of soluble food. The spots left by the drops regurgitated from the crops are "vomit spots" and are light in colour with dark centres. Fæcal spots can readily be distinguished from vomit spots, they are opaque, and white or yellow in colour. An average of fifteen to twenty spots are produced by a fly in twenty-four hours, in the proportion of one fæcal to four or six vomit.

### Carriers of Disease.

The house-fly has been proved to carry the following disease germs: Enteric fever, infantile diarrhœa, tuberculosis, diphtheria, ophthalmia, anthrax, cholera, and plague. The range of flight varies from 300 to 1,700 yards from the point of liberation. They fly with the wind, and a greater distance in open country than in towns. Their habit is to remain in the vicinity of their birthplace, and, provided food is plentiful, very few reach the extreme range. They may, however, travel for many miles on animals or men.

Flies carry the infective germs, which they acquire from the filth on which they feed, on their hairy legs and bodies and in their intestines.

Their legs are never free from organisms, seldom from *Staphylococcus pyogenes aureus* and *Bacillus coli.* Germs, however, do not survive on them for long, as they are frequently brushed away, but re-infection is constant. Owing to the persistence of bacteria in the crop and intestines, infection from the alimentary tract is the usual method, either by the regurgitated vomit or by the fæcal spots. Graham Smith fed flies with various bacillary emulsions, with the results shown in the following table, in which the column "Number of Days" indicates the periods during which the bacilli were recoverable from the intestinal tract:

| Germ | Number of Days Crop | Intestines |
|---|---|---|
| Tubercle .. .. ... | 3 | 12 to 16 |
| Diphtheria ... ... .. | 2 | 2 |
| Cholera .. .. .. | 2 | 2 |
| *Bacillus enteritidis* .. .. | 7 | 7 |
| Anthrax, spores .. .. | 20 | 20 |
| „ bacilli ... .. | 7 | 3 |

Anthrax bacilli were recovered from the crops and intestines of dead flies for 115 days.

### Open Latrines are Dangers to Health.

Experiments conducted under natural conditions are more convincing than those with pure cultures *in vitro*, and the most valuable of all are those by Faichnie in India in 1909, which demonstrated that flies bred in enteric excreta were born containing enteric bacilli in their intestines, and that, after excluding re-infection, by feeding on sterile sugar and water, these germs were recoverable from their excrement in varying periods up to the sixteenth day. Further, during the investigation of outbreaks of enteric fever he recovered the enteric bacillus from the intestines of flies captured in the verandahs of artillery and infantry cookhouses,

☞ Previous articles appeared on Feb. 9, 23, March 9, 23, April 6, 20, and May 4 and 25, p. 157.

**THE GREAT WAR**—(*continued*).

in the bungalow of an officer suffering from the disease, in an officers' mess, in a Royal Artillery coffee-shop, and on a night soil trenching ground. Enteric bacilli have also been isolated from naturally infected flies by Ficker in Leipzig and by Hamilton in Chicago.

Although not definitely proved, circumstantial evidence points very strongly to a causative connection between flies and summer diarrhœa, epidemics of which coincide with their activity and circulation. Morgan has isolated a bacillus from a very large percentage of cases of infantile diarrhœa, which produced fatal diarrhœa in rats and monkeys, and which was recovered from flies caught in infected and uninfected houses. Flies feed voraciously on tuberculous sputum, which produces diarrhœa in them, and, consequently, a large number of fæcal spots, and, as the bacilli are recoverable from them up to sixteen days, there is ample opportunity for them to be deposited on our food or disseminated, after desiccation, in the air. The prevention of the access of flies to sputum of any kind is clearly indicated.

BREEDING-PLACES.

Breeding-places have the most important bearing on flies in their relation to the spread of disease, for, if we prevent breeding, we prevent flies. In the Army area in France there is only one class of breeding material which produces them in great numbers—viz. animal excreta, mainly horse and mule dung. They also breed in rabbit-hutches, which are to be found in every farm-house, in pig-styes, fowl-runs, slaughter-yards, and brewery waste. The most prolific source is the farm *fumier*, or manure midden, a large dung-pit, from 6 to 10 feet deep, to be found in every farm-yard. In it the stable litter is deposited daily and allowed to ferment and mature until the spring, when it is applied to the land.

Flies lay their eggs in dung as soon after it is deposited as opportunity offers. The chance of its being seeded lessens from this moment, and as soon as it is dry on the surface it ceases to be attractive, for the larvæ when hatched could not penetrate the hardened crust. Oviposition does not occur in fermented or mouldy litter; the larvæ require soft, fresh food, a warm temperature, and persistent moisture of the breeding material. The warmest months are the most prolific. There is a fourteen-fold increase in August compared with July.

As soon as dung has been seeded the mischief has been done, whether it is allowed to remain where it fell or stored in closed bins or in open pits, or carted away to manure heaps, the eggs accompany it, their development progressing, accelerated by heat or retarded by cold, until the pupa stage is reached, in which they rest indefinitely, dependent for their complete development on the temperature and a means of escape.

(*To be continued.*)

